# Blood sampled from existing peripheral IV cannulae yields results equivalent to venepuncture: a systematic review

**DOI:** 10.1177/2054270419894817

**Published:** 2020-05-06

**Authors:** Finnian D Lesser, David A Lanham, Daniel Davis

**Affiliations:** 1 Acute Medical Unit, Conquest Hospital, Hastings, East Sussex Healthcare Trust, Saint Leonards-on-sea TN37 7RD, UK; 2 MRC Unit for Lifelong Health and Ageing at UCL and Acute Medical Unit, University College London NHS Foundation Trust, London NW1 2BU, UK

**Keywords:** Venepuncture, blood sampling, peripheral venous cannula, phlebotomy

## Abstract

**Objectives:**

To establish whether blood samples taken from used peripheral intravenous cannulae are clinically interchangeable with venepuncture.

**Design:**

Systematic review. PubMed, Web of Science and Embase were searched for relevant trials.

**Setting:**

Trials which compared blood samples from used peripheral intravenous cannulae to venepuncture and provided limits of agreement or data which allowed calculation of limits of agreement.

**Participants:**

Seven trials with 746 participants. Blood tests included 13 commonly ordered biochemistry, haematology and blood gas measurements.

**Main outcome measures:**

95% limits of agreement. Data were pooled using inverse variance weighting and compared to a clinically acceptable range estimated by expert opinion from previous trials.

**Results:**

Limits of agreement for blood samples from used peripheral intravenous cannulae were within the clinically acceptable range for sodium, chloride, urea, creatinine and haematology samples. Limits of agreement for potassium were ±0.47 mmol/L which exceeded the clinically acceptable range. Peripheral intravenous cannula samples for blood gas analysis gave limits of agreement which far exceeded the clinically acceptable range.

**Conclusions:**

Blood sampling from used peripheral intravenous cannulae is a reasonable clinical practice for haematology and biochemistry samples. Potassium samples from used peripheral intravenous cannulae can be used in situations where error up to ±0.47 mmol/L is acceptable. Peripheral intravenous cannula samples should not be used for blood gas analysis.

## Introduction

Venepuncture carries a certain amount of pain and a small risk of complications. Given many patients have an *in-situ* peripheral intra-venous cannula, sampling blood from this obviates the need for repeated venepuncture if there are clinically equivalent and reliable results. In general, this is not common practice due to concerns regarding the validity of results.^1^ Other considerations include the method used for obtaining samples, prior or concurrent use of the cannula for fluid administration, the aspiration volume needing to be discarded and any need for special equipment.

Whether assay results from peripheral intravenous cannula and venepuncture are clinically equivalent is quantified using limits of agreement, expressed as the range within which 95% of values will lie in comparison to a reference standard measurement.^2^ The limits of agreement are then compared with a clinically acceptable range usually defined by consensus.

In order to assess the quantity and quality of studies comparing blood samples from existing PIV with venepuncture, we undertook a systematic review. We focused on studies of sufficient quality to influence clinical practice.

## Methods

### Search strategy

**Figure 1.**
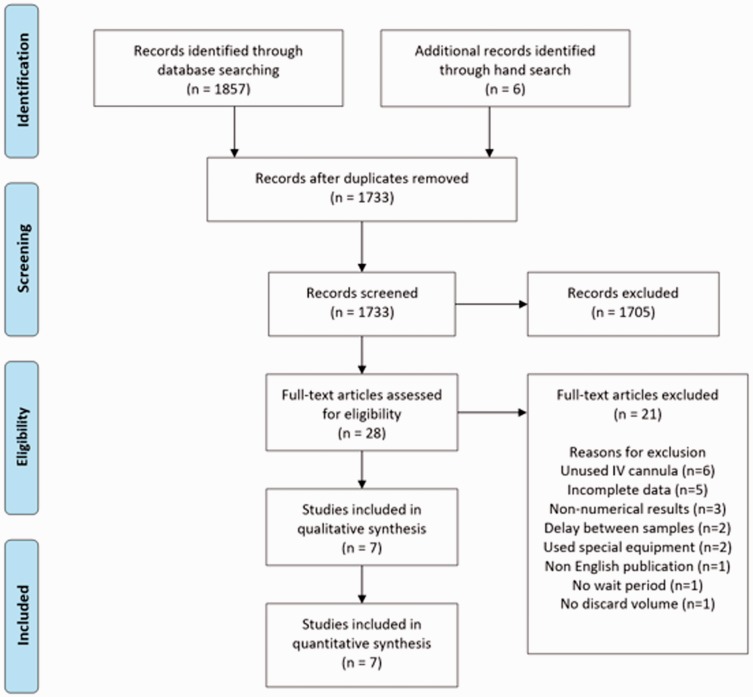
Flow diagram showing search strategy. Source: Adapted from PRISMA flow diagram.^11^

Relevant keywords and terms were developed through a scoping search in PubMed, eventually expanding to three databases (PubMed, ISI Web of Science and Embase). Two reviewers screened titles and abstracts (FL and DL). The abstracts were categorised into ‘not relevant’ and ‘potentially relevant’ and all ‘potentially relevant’ studies were reviewed in full. References of included studies were also hand searched (Figure 1). The full search strategy for PubMed is shown in Appendix 1.

### Study selection

#### Inclusion criteria

1. Studies that compared human blood samples drawn from peripheral intravenous cannulae and venepuncture.
2. Studies reporting numerical results for at least one of the following tests: sodium, potassium, chloride, urea (or blood urea nitrogen), creatinine, haemoglobin, haematocrit, white cell count, platelets, international normalised ratio, pH, partial pressure oxygen, partial pressure carbon dioxide.
3. Studies using the Bland–Altman method for limits of agreement or providing data which allowed the calculation of limits of agreement.^2^

#### Exclusion criteria

1. Articles not in English.
2. Studies which used newly inserted peripheral intravenous cannula for blood sampling unless intravenous fluids had been infused through the cannula prior to sampling.
3. Studies which took samples while infusions were running through the cannula or did not wait after stopping infusions.
4. Delay of greater than 5 min between samples for comparison.
5. Studies which did not discard at least 2 mL of aspirate prior to blood sampling.
6. Studies which required special equipment for blood sampling from peripheral intravenous cannula (for example double stopcock techniques).

### Data collection and extraction

Relevant data were extracted from included papers in duplicate (FL and DL), including publication year, patient population, number of patients, blood tests carried out, discard volume prior to sampling, wait time between stopping infusions and sampling, aspiration method and cannula gauge as well as the assay results. Tests measured in non-SI units were converted to SI units. Blood urea nitrogen results were converted to urea by multiplying by 2.14 and then converting to SI units.

The 95% limits of agreement was the primary outcome of interest. If not reported directly, it was calculated

$$\begin{array}{c} \text{Limits}\;\text{of}\;\text{agreement}=\text{Meandifference}\pm 1.96\\ \times \text{SDofdifferencesfrommean} \end{array}$$

Limits of agreement values were then pooled using inverse variance weighting

$$\begin{array}{c} {\text{variance}}_{\left(\text{pooled}\right)}\\ =\frac{{(\text{variance}}_{\text{study}1}\times {\text{n}}_{\text{study}1})+{(\text{variance}}_{\text{study}2}\times {\text{n}}_{\text{study}2})\ldots }{{(\text{n}}_{\text{study}1}-1)+{(\text{n}}_{\text{study}2}-1)\ldots } \end{array}$$

### Clinically acceptable limits of agreement

The clinically acceptable errors in blood sampling are not fully established and vary depending on patient situation and the clinicians’ tolerance for error. Four studies specified such ranges established through clinician survey.^3–6^ We used a mean of these values to define clinically acceptable limits for this review.

## Results

Literature search provided 1857 articles for abstract review with 130 duplicates (Figure 1). Hand-searching identified a further six studies for abstract review. There were 21 papers which were excluded at full text review for failure to meet the inclusion criteria or meeting the exclusion criteria (Appendix 2). Ultimately, seven studies were included with total individuals n = 746 from a combination of adult inpatient, adult emergency department, paediatric inpatient and healthy volunteers (Table 1).

**Table 1.** Summary of included studies.

Year	First author	n	Population	Infusions prior to blood sampling	Discard volume (mL)	Wait time after stopping infusion	Sampling technique	PIV gauge (French)
1998	Fincher	53	Medical inpatient adults	Not specified	3	60 min	Syringe	18, 20
2001	Himberger	64	Emergency department adults	At least 100 mL	5	1 min	Syringe and needle	16,18,20
2001	Zlotowski	33	Healthy volunteers	200 mL normal saline	12	2 min	Syringe and needle	18
2007	Corbo	81	Emergency department adults	Not stated	5	2 min	Vacutainer and needleless adapter	18, 20, 22
2010	Berger-Achituv	40	Inpatient paediatrics	At least 100 mL	2	1 min	Syringe	20, 22, 24
2014	Ortells-Abuye	272	Inpatient ward or short stay unit adults	Medications or IV fluids	4	15 s	Syringe	16, 18, 20, 22
2014	Hambleton	259	Emergency department adults	Unspecified	2	2 min	Vacutainer with luer adapter	18, 20

### Characteristics of included studies

Studies comparing venepuncture to peripheral intravenous cannula used different methods and protocols (Table 1). The minimum discard volume in the studies was 2 mL. Cannula sizes varied from 16 to 22 French. All cannulae had been used prior to sampling but there was variation in volume and contents of infusion prior to sampling. Sampling devices used were either syringe or vacutainer systems.

### Biochemistry

Sodium, chloride, urea and creatinine pooled limits of agreement were all within the clinically acceptable error range (Table 2). In some cases, the pooled limits of agreement was substantially lower than the clinically acceptable error range (e.g. creatinine in µmol/L limits of agreement = −13, +12; clinically acceptable range = ±26). However, potassium limits of agreement exceeds the clinically acceptable error range (in mmol/L limits of agreement = −0.48, +0.46; clinically acceptable range = ±0.35).

**Table 2.** Results for renal function and electrolytes. Showing 95% limits of agreement for included papers, pooled 95% limits of agreement and clinically accepted range for comparison.

Study	Sodium (mmol/L)		Potassium (mmol/L)		Chloride (mmol/L)		Urea (mmol/L)		Creatinine (µmol/L)	
	Lower bound	Upper bound	Lower bound	Upper bound	Lower bound	Upper bound	Lower bound	Upper bound	Lower bound	Upper bound
Fincher			−0.47	0.45						
Himberger	−2.5	3.3	−0.45	0.56	−3.3	3.9	−0.5	0.6	−16	18
Zlotowski	−2.2	2.7	−0.23	0.39	−1.9	1.8	−0.6	0.7	−8.7	8.7
Corbo	−4.0	3.0	−0.70	0.70	−3.3	2.9				
Berger-Achituv	−3.3	2.9	−0.42	0.43	−3.7	2.9	−0.9	0.8		
Ortells-Abuye	−2.6	3.1	−0.45	0.45			−1.1	1.2		
Hambleton	−3.5	3.6	−0.46	0.40	−2.6	3.0	−0.6	0.6	−13	11
**Pooled results**	**−2.9**	**3.3**	**−0.48**	**0.46**	**−2.8**	**3.0**	**−0.8**	**0.9**	**−13**	**12**
**Clinically acceptable range**	**−4.3**	**4.3**	**−0.35**	**0.35**	**−6.5**	**6.5**	**−1.1**	**1.1**	**−26**	**26**

### Haematology and international normalised ratio

The pooled results for haematology and international normalised ratio were all within the clinically acceptable limits of agreement (Table 3).

**Table 3.** Results for haematology and international normalised ratio (INR). Showing 95% limits of agreement for included papers, pooled 95% limits of agreement and clinically accepted range for comparison.

Study	Haemoglobin (g/dL)		Haematocrit (%)		White cells (1000/L)		Platelets (1000/L)		INR	
	Lower bound	Upper bound	Lower bound	Upper bound	Lower bound	Upper bound	Lower bound	Upper bound	Lower bound	Upper bound
Fincher			−0.6	0.9						
Himberger	−0.70	0.70	−1.4	1.3	−0.8	0.7	−30	27		
Zlotowski	−0.34	0.40	−1.0	1.2	−0.7	0.4	−13	13	−0.06	0.06
Corbo	−0.61	0.91	−1.4	1.7						
Berger-Achituv	−0.66	0.57	−2.1	1.9	−1.1	1.1	−37	39		
Ortells-Abuye	−0.65	0.65			−1.8	1.8	−30	34		
Hambleton	−0.58	0.47	−0.6	0.5	−0.9	0.8	−19	16	−0.12	0.11
**Pooled results**	**−0.63**	**0.57**	**−0.74**	**0.64**	**−1.3**	**1.2**	**−25**	**26**	**−0.12**	**0.10**
**Clinically acceptable range**	**−0.9**	**0.9**	**−3.5**	**3.5**	**−1.5**	**1.5**	**−40**	**40**	**−0.20**	**0.20**

**Table 4.** Results for blood gas and pH. Showing 95% limits of agreement for included papers, pooled 95% limits of agreement and clinically accepted range for comparison.

Study	pH		pO_2_ (kPa)		pCO_2_ (kPa)	
	Lower bound	Upper bound	Lower bound	Upper bound	Lower bound	Upper bound
Ortells-Abuye	−0.060	0.040	−5.4	2.9	−0.8	1.4
Hambleton	−0.034	0.044	−2.9	3.8	−1.2	0.9
**Pooled results**	**−0.050**	**0.040**	**−4.5**	**3.0**	**−0.9**	**1.3**
**Clinically acceptable range**	**−0.1**	**0.1**	**−0.7**	**0.7**	**−0.7**	**0.7**

### Blood gases and pH

The pooled limits of agreement for pH was within clinically acceptable range (Table 4). However, the pooled limits of agreement for pCO_2_ exceeded the clinically acceptable range (in kPa limits of agreement = −0.9, +1.3; clinically acceptable range = ±0.7) and limits of agreement pO_2_ dramatically exceeded the clinical acceptable range (in kPa limits of agreement = −4.5, +3.0; clinically acceptable range = ±0.7).

## Discussion

Across pooled studies, we showed that assays from used peripheral intravenous cannula were reliable and clinically consistent with fresh venepuncture samples, except in the case of potassium and blood gases. Taken together, our findings suggest that peripheral intravenous cannula sampling could be given greater consideration in clinical practice – at least for the tests described.

The results for potassium levels were not within clinically acceptable agreement limits for some patients. The 95% limits of agreement for potassium of −0.46 to + 0.47 mmol/L shows that, for patients where a tight control of potassium is essential, samples from used peripheral intravenous cannula should be used with caution. For most other patients, a sample from a peripheral intravenous cannula would be sufficient and the level of error ±0.47 mmol/L is unlikely to affect patient outcomes. The cause for the higher level of error is not clear, though haemolysis or haemodilution was excluded as causes in the studies considered here. For blood gas analyses (pO_2_ and pCO_2_), the two studies reporting these suggested errors were due to contamination of the samples with atmospheric air post-collection.^6,7^

There were some limitations to this study. There was heterogeneity in study populations, protocols and equipment. It is not clear whether our findings are generalisable to other sampling techniques, e.g. with narrower gauge cannula.

In terms of haemolysis degrading the sample quality, we show that for most blood tests it does not lead to significant errors. However, if the laboratory or analysers do not check for haemolysed samples it could lead to errors in results.^8^

We did not assess some commonly ordered blood tests. Our findings relate to specific assays and may not be generalisable to other haematology and biochemistry investigations.

The clinical impact of these findings will be greatest in those situations in which patients require repeated blood tests where samples from peripheral intravenous cannula would be suitable. For example, if a patient were admitted with symptomatic anaemia and needed serial haemoglobin measurements samples from a cannula could be used. Peripheral intravenous cannula sampling can be an alternative for patients who find venepuncture intensely distressing. There are also patients in which venepuncture is technically difficult and peripheral intravenous cannula samples can provide easier access to blood.

Our findings do not explain why some blood tests are not reliable when taken from a used peripheral intravenous cannula and this could be the subject of further research. Further studies could also be considered to assess other assays which were not included in this paper.

## Conclusions

Peripheral intravenous cannula samples are interchangeable with venepuncture for sodium, chloride, urea, creatinine and haematology tests. Peripheral intravenous cannula samples can be used for potassium measurement in situations where error of ±0.47 mmol/L is acceptable. Blood gas analysis for pO_2_ and pCO_2_ can show clinically significant differences between peripheral intravenous cannula and venepuncture and so peripheral intravenous cannula samples should not be used. Overall, peripheral intravenous cannula sampling is a reasonable clinical practice for a range of common assays.

## Declarations

### Competing Interests

None declared.

### Funding

DD is supported by a Wellcome Trust Intermediate Clinical Fellowship (WT107467). DL is supported as a UCLH CEO clinical research fellow.

### Ethics approval

Not applicable.

### Guarantor

FL.

### Contributorship

FL and DL both conceived the research. FL led the planning of the research. FL and DL contributed equally to the literature search, review of literature and data analysis. All authors were involved in drafting and have approved the final version. FL is the guarantor and principal investigator, accepting responsibility for the study.

## Supplemental Material

sj-pdf-1-shr-10.1177_2054270419894817 - Supplemental material for Blood sampled from existing peripheral IV cannulae yields results equivalent to venepuncture: a systematic review Click here for additional data file. Supplemental material, sj-pdf-1-shr-10.1177_2054270419894817 for Blood sampled from existing peripheral IV cannulae yields results equivalent to venepuncture: a systematic review by Finnian D Lesser, David A Lanham and Daniel Davis in JRSM Open

## Appendix 1. PubMed search strategy

1. ((cannula) OR saline lock device) OR peripheral venous catheter OR (Catheterization, Peripheral*) OR Infusions, Intravenous/instrumentation*
2. ((blood sampling) OR Blood collection) OR phlebotomy
3. Humans[MeSH Terms]
4. #1 AND #2 AND #3
