# Supplementary material for: Blood sampled from existing peripheral IV cannulae yields results equivalent to venepuncture: a systematic review
Source: JRSM Open. 2020 May 6;11(5):2054270419894817. doi: 10.1177/2054270419894817 (PMC7236571; doi:10.1177/2054270419894817)
Supplement: sj-pdf-1-shr-10.1177_2054270419894817 - Supplemental material for Blood sampled from existing peripheral IV cannulae yields results equivalent to venepuncture: a systematic review [file sj-pdf-1-shr-10.1177_2054270419894817.pdf]

QUOROM Statement checklist

Heading	Subheading	Descriptor	Reported? (Y/N)	Page Number
**Title**		Identify the report as a systematic review	Y	1
**Abstract**		Use a structured format	Y	2
	Objectives	The clinical question explicitly	Y	2
	Data sources	The databases (ie, list) and other information sources	Y	2
	Review methods	The selection criteria (ie, population, intervention, outcome, and study design); methods for validity assessment, data abstraction, and study characteristics, and quantitative data synthesis in sufficient detail to permit replication	Y	2
	Results	Characteristics of the RCTs included and excluded; qualitative and quantitative findings (ie, point estimates and confidence intervals); and subgroup analyses	Y	2
	Conclusion	The main results	Y	2
		**Describe**		
**Introduction**		The explicit clinical problem, biological rationale for the intervention, and rationale for review	Y	2
**Methods**	Searching	The information sources, in detail (eg, databases, registers, personal files, expert informants, agencies, hand-searching), and any restrictions (years considered, publication status, language of publication)	Y	2
	Selection	The inclusion and exclusion criteria (defining population, intervention, principal outcomes, and study design	Y	2
	Validity assessment	The criteria and process used (eg, masked conditions, quality assessment, and their findings)	Y	4 (Table1)
	Data abstraction	The process or processes used (eg, completed independently, in duplicate)	Y	3
	Study characteristics	The type of study design, participants' characteristics, details of intervention, outcome definitions, and how clinical heterogeneity was assessed	Y	3
	Quantitative data synthesis	The principal measures of effect (eg, relative risk), method of combining results (statistical testing and confidence intervals), handling of missing data; how statistical heterogeneity was assessed; a rationale for any a-priori sensitivity and subgroup analyses; and any assessment of publication bias	Y	3
**Results**	Trial flow	Provide a meta-analysis profile summarising trial flow (see figure)	Y	Figure 1
	Study characteristics	Present descriptive data for each trial (eg, age, sample size, intervention, dose, duration, follow-up period)	Y	5 (Table 1)
	Quantitative data synthesis	Report agreement on the selection and validity assessment; present simple summary results (for each treatment group in each trial, for each primary outcome); present data needed to calculate effect sizes and confidence intervals in intention-to-treat analyses (eg 2X2 tables of counts, means and SDs, proportions)	Y	Tables 2- 4
**Discussion**		Summarise key findings; discuss clinical inferences based on internal and external validity; interpret the results in light of the totality of available evidence; describe potential biases in the review process (eg, publication bias); and suggest a future research agenda	Y	4
